# Puerarin enhances intestinal function in piglets infected with porcine epidemic diarrhea virus

**DOI:** 10.1038/s41598-021-85880-5

**Published:** 2021-03-22

**Authors:** Mengjun Wu, Dan Yi, Qian Zhang, Tao Wu, Kui Yu, Meng Peng, Lei Wang, Di Zhao, Yongqing Hou, Guoyao Wu

**Affiliations:** 1 Hubei Key Laboratory of Animal Nutrition and Feed Science, Wuhan Polytechnic University, Wuhan, 430023 China; 2 Department of Animal Science, Texas A&M University, College Station, TX 77843 USA; 3 Department of Agricultural, Environmental and Food Sciences, University of Molise, 86100 Campobasso, Italy

**Keywords:** Drug discovery, Microbiology, Molecular biology

## Abstract

Puerarin has been reported to be an excellent antioxidant, anti-inflammatory and antimicrobial agent, but the potential effect of puerarin on porcine epidemic diarrhea virus (PEDV) is unclear. This study aimed to determine whether puerarin could alleviate intestinal injury in piglets infected with PEDV. A PEDV (Yunnan province strain) infection model was applied to 7-day-old piglets at 10^4.5^ TCID_50_ (50% tissue culture infectious dose). Piglets were orally administered with puerarin at the dosage of 0.5 mg/kg body weight from day 5 to day 9. On day 9 of the trial, piglets were inoculated orally with PEDV. Three days later, jugular vein blood and intestinal samples were collected. Results showed puerarin reduced morbidity of piglets infected with PEDV. In addition, puerarin reduced the activities of aspartate aminotransferase and alkaline phosphatase, the ratio of serum aspartate aminotransferase to serum alanine aminotransferase, the number of white blood cells and neutrophils, and the plasma concentrations of interleukin-6, interleukin-8 and tumor necrosis factor-α, as well as protein abundances of heat shock protein-70 in PEDV-infected piglets. Moreover, puerarin increased D-xylose concentration but decreased intestinal fatty acid-binding protein concentration and diamine oxidase activity in the plasma of piglets infected with PEDV. Puerarin increased the activities of total superoxide dismutase, glutathione peroxidase and catalase, while decreasing the activities of myeloperoxidase and concentration of hydrogen peroxide in both the intestine and plasma of PEDV-infected piglets. Puerarin decreased mRNA levels of glutathione S-transferase omega 2 but increased the levels of nuclear factor erythroid 2-related factor 2*.* Furthermore, puerarin increased the abundance of total eubacteria (16S rRNA)*, Enterococcus* genus*, Lactobacillus* genus and *Enterobacteriaceae* family in the intestine, but reduced the abundance of *Clostridium coccoides* in the caecum. These data indicate puerarin improved intestinal function in piglets infected by PEDV and may be a promising supplement for the prevention of PEDV infection.

## Introduction

Some isoflavonoids are considered to be beneficial to human health partially due to their antioxidant properties^1^. Puerarin (PR), an isoflavone extracted from *kudzu* root, possesses neuroprotective and antioxidant properties. PR was reported to inhibit nitric oxide (NO) and intracellular ROS production induced by lipopolysaccharide in N9 microglial cells^2^ and to decrease the activities of caspase-3 and caspase-9 in PC12 cells, thereby protecting cells from oxidative injury^3^. It has been reported that PR has anti-inflammatory activity in an inflammatory cell model^4^. PR can partly attenuate the detrimental inflammation induced by cerebral ischemia/reperfusion by activating the cholinergic anti-inflammatory pathway^5^. In addition, PR may be used as an antimicrobial agent^6^ since PR could protect porcine intestinal epithelial cells (IPEC-J2) against enterotoxigenic *Escherichia coli* (ETEC) infection through inhibiting bacterial adhesion and inflammatory responses^7^. The latest research found that PR could suppress the rise of mRNA levels of porcine epidemic diarrhea virus (PEDV) N and M genes in intestines of PEDV-infected piglets^8^.

Porcine epidemic diarrhea (PED), caused by PEDV, is an intestinal infectious disease characterized by vomiting, anorexia, watery diarrhea, and dehydration^9^, which leads to dramatic mortality in neonatal piglets and is, therefore, an overwhelming threat to the swine industry worldwide^10^. The latest research, as provided evidence for airborne transmission of PEDV^11^, demonstrated greater transmission potential of PEDV than that of other seasonal diarrhea viruses^12^. The small intestine, especially the jejunum and ileum, is the target of PEDV^13^. An intact intestinal barrier plays a vital role in preventing the virus, bacteria and dietary allergens from entering the mucosa^14,15^. In the gut, the microbiota is an important biological barrier that prevents invaders from entering the body^16^. Specifically, the composition of the small intestinal microbiota is markedly altered in sucking piglets infected with PEDV^17^. After PEDV infection, the most predominant changes are the reduced expression of proteins related to oxidative stress, and the enhanced expression of proteins involved in inflammatory responses^9^. Notably, among the PEDV strains, virulent strain such as CH/YNKM-8/2013 can strongly activate the NF-κB pathway and caused much more intensive inflammatory cascades than attenuated vaccine CV777^18^.

As a traditional Chinese herb, PR shows excellent antioxidant, anti-inflammatory and antimicrobial properties. Results of our previous study indicated that PR attenuated the reduction of cell proliferation in vitro, and inhibited PEDV replication and the expression of several cytokines^8^. Considering the good antioxidant property of PR and the special role of intestinal bacteria, we hypothesized that PR could alleviate PEDV-induced intestinal oxidative stress and inflammation, and modulate the intestinal flora in piglets. This study was conducted to test the hypothesis and elucidate the underlying mechanisms. We are not aware of publications that report the roles of PR in alleviating intestinal injury and modulating intestinal microbes in PEDV-challenged piglets. The findings of this study will provide new perspectives for PR in preventing or mitigating PEDV in swine.

## Results

### Clinical observations and body weight in PEDV-infected piglets

**Figure 1:**
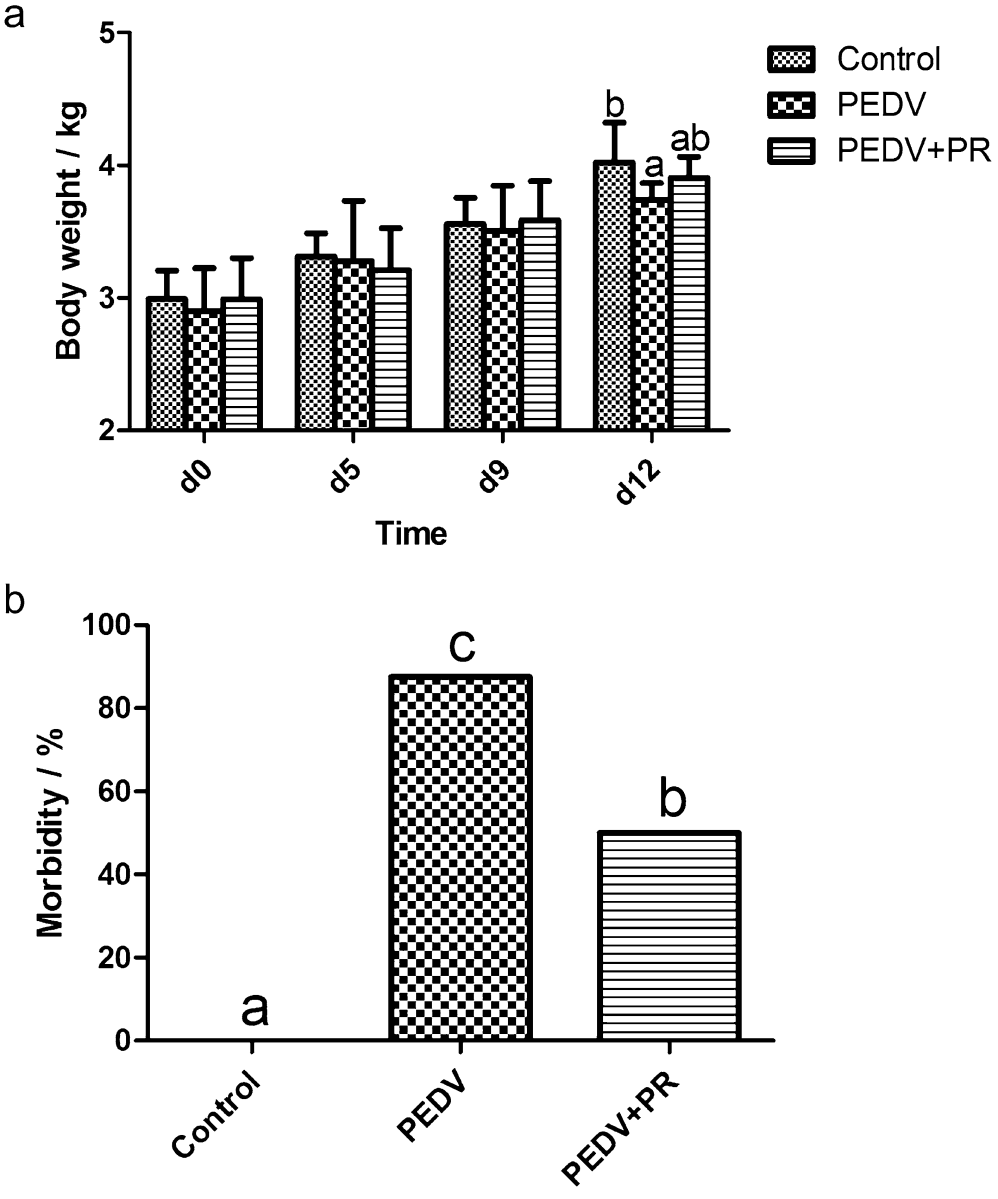
Effects of puerarin (PR) administration on body weight and morbidity of piglets after PEDV infection. (**a**) body weight. (**b**) morbidity. Values are mean and SD, n = 8. ^a, b, c^, Values within a column not sharing a common superscript letter indicate significant difference at *P* < 0.05.

PEDV infection significantly reduced the body weight and increased morbidity of piglets as compared with the control (*P* < 0.05), while PR administration reduced the morbidity of PEDV- infected piglets (Fig. 1).

### Plasma biochemical parameters and blood cell counts

Data on plasma biochemical parameters and blood cell counts are summarized in Table 1. Compared with non-infected pigs, PEDV-infected pigs had higher activities of aspartate aminotransferase (AST) and alkaline phosphatase (ALP), total cholesterol (CHOL) level, and AST/ALT ratio, but lower alanine aminotransferase (ALT) activity and levels of total bilirubin (TBIL), triglyceride (TG), and urea in the plasma (*P* < 0.05). However, PR-administered piglets exhibited lower activities of AST and ALP and lower concentrations of CHOL in plasma, but higher activity of ALT and higher concentrations of TBIL, TG and urea in plasma, compared with PEDV-infected pigs without receiving PR (*P* < 0.05). PEDV increased the numbers of white blood cells (WBC), neutrophils (NEU), lymphocytes (LYM) and red blood cells (RBC), while decreasing the numbers of monocytes (MONO), monocyte ratio (MONOR), mean platelet volume (MPV), and platelet distribution width ratio (PDWR) in the blood (*P* < 0.05). In addition, PR administration reduced the numbers of WBC, NEU and RBC, but elevated the numbers of MONO, MONOR, MPV and PDWR (*P* < 0.05) in comparison with PEDV-infected pigs.

**Table 1:** Effects of puerarin (PR) administration on plasma biochemical parameters and immune cell numbers in piglets infected with PEDV.

Items	Control	PEDV	PEDV + PR
**Plasma biochemical parameters**			
ALT (U/L)	75.6 ± 4.35^b^	63.5 ± 5.66^a^	75.2 ± 8.84^b^
AST (U/L)	43.3 ± 8.35^a^	55.6 ± 8.77^b^	46.3 ± 3.56^a^
AST/ALT	0.63 ± 0.14^a^	0.79 ± 0.16^b^	0.67 ± 0.19^ab^
Total bilirubin (μmol/L)	11.1 ± 4.22^b^	6.62 ± 0.84^a^	12.3 ± 6.86^b^
Total protein (g/L)	54.2 ± 3.45	52.2 ± 1.98	51.7 ± 3.02
Albumin (g/L)	29.6 ± 2.22	28.0 ± 1.25	28.6 ± 2.58
Cholesterol (mmol/L)	2.22 ± 0.42^a^	2.95 ± 0.23^b^	2.42 ± 0.30^a^
Triglyceride (mg/dL)	0.72 ± 0.14^b^	0.50 ± 0.12^a^	0.72 ± 0.21^b^
Urea nitrogen(mmol/L)	4.11 ± 1.56^b^	1.13 ± 0.15^a^	3.41 ± 1.58^b^
ALP (U/L)	356 ± 91.2^a^	439 ± 78.6^b^	345 ± 74.6^a^
Creatinine (µmol/L)	73.8 ± 8.05	75.8 ± 9.27	77.5 ± 5.30
Glucose (mmol/L)	5.01 ± 0.57	5.28 ± 0.39	5.00 ± 0.85
GGT (mmol/L)	43.3 ± 8.27	41.4 ± 9.52	41.6 ± 12.8
**Blood cell counts**			
White blood cells (10^9^/L)	8.85 ± 2.04^a^	12.9 ± 1.99^b^	9.97 ± 3.40^a^
Neutrophils (10^9^/L)	3.45 ± 1.02^a^	5.56 ± 0.18^b^	3.45 ± 1.09^a^
Lymphocytes (10^9^/L)	4.86 ± 1.17^a^	6.96 ± 1.10^b^	5.91 ± 2.29^ab^
Monocytes (10^9^/L)	0.34 ± 0.08^b^	0.21 ± 0.07^a^	0.36 ± 0.16^b^
Monocyte ratio (%)	3.37 ± 1.07^b^	2.03 ± 0.71^a^	3.09 ± 0.91^b^
Red blood cells (10^12^/L)	5.95 ± 0.49^a^	6.53 ± 0.42^b^	6.11 ± 0.23^a^
MPV (fL)	9.24 ± 0.72^b^	8.09 ± 0.86^a^	8.99 ± 0.49^b^
PDWR (%)	68.6 ± 4.06^b^	61.8 ± 4.91^a^	67.6 ± 4.68^b^

Values are mean and SD, *n* = 8.

^a,b^Within a row, means with different superscripts differ (< 0.05).

ALT, alanine aminotransferase; AST, aspartate aminotransferase; ALP, alkaline phosphatase; GGT, γ-glutamyltransferase; MPV, mean platelet volume; PDWR, Platelet distribution width ratio.

### DAO activity, D-xylose and I-FABP concentrations in plasma

**Figure 2:**
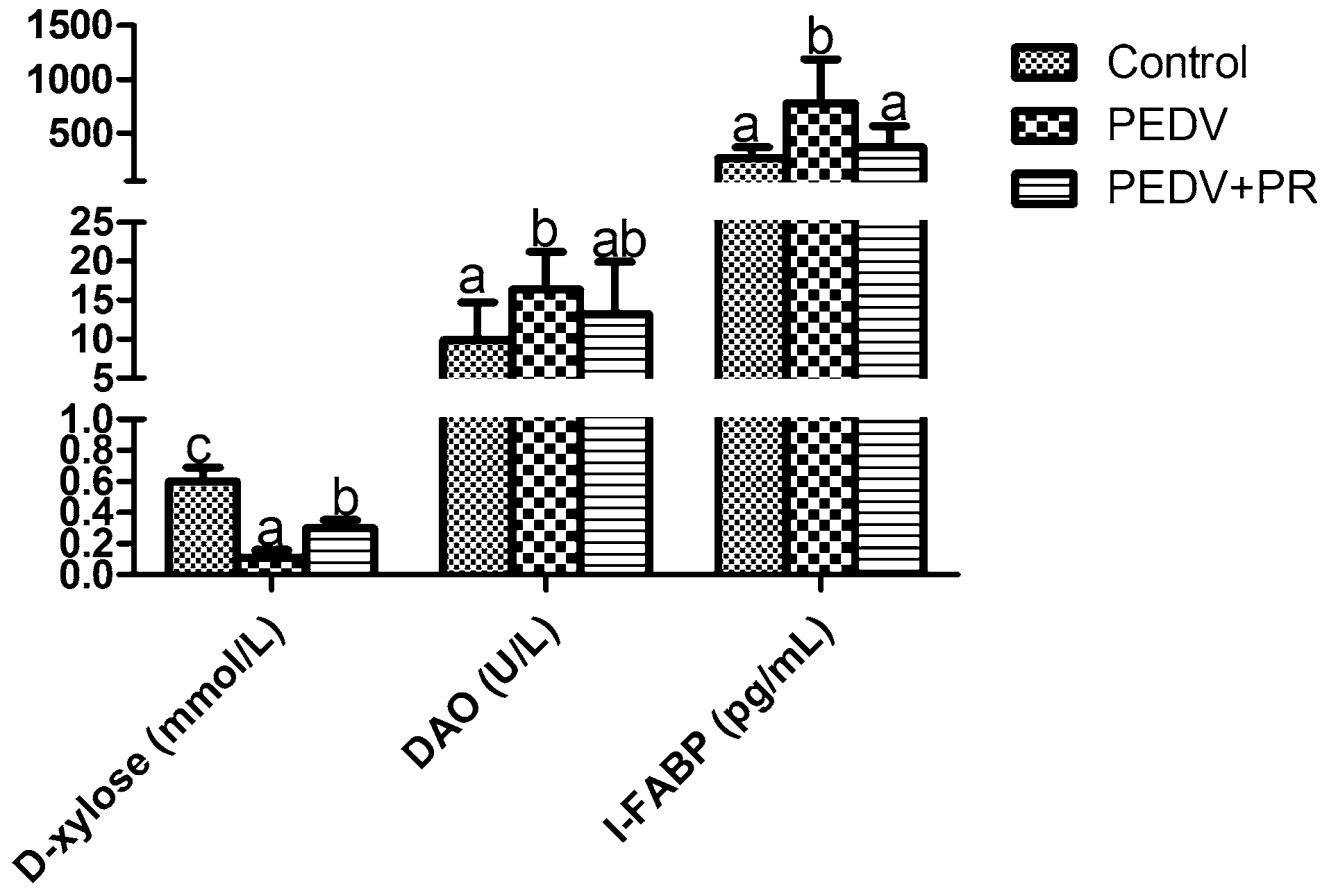
Effects of puerarin (PR) administration on blood DAO activity and D-xylose, I-FABP concentrations of piglets after PEDV infection. Values are mean and SD, *n* = 8. ^a, b, c^, Values within a column not sharing a common superscript letter indicate significant difference at *P* < 0.05.

Data on blood DAO activity, D-xylose and I-FABP concentrations are summarized in Fig. 2. PEDV infection decreased D-xylose concentration in plasma, but increased I-FABP concentration and DAO activity in plasma, when compared with the control *(P* < 0.05). On the contrary, PR administration to PEDV-infected piglets increased D-xylose concentration in plasma, but decreased I-FABP concentration (*P* < 0.05).

### Anti-oxidative enzymes and oxidation-relevant products in plasma and intestinal mucosae

**Figure 3:**
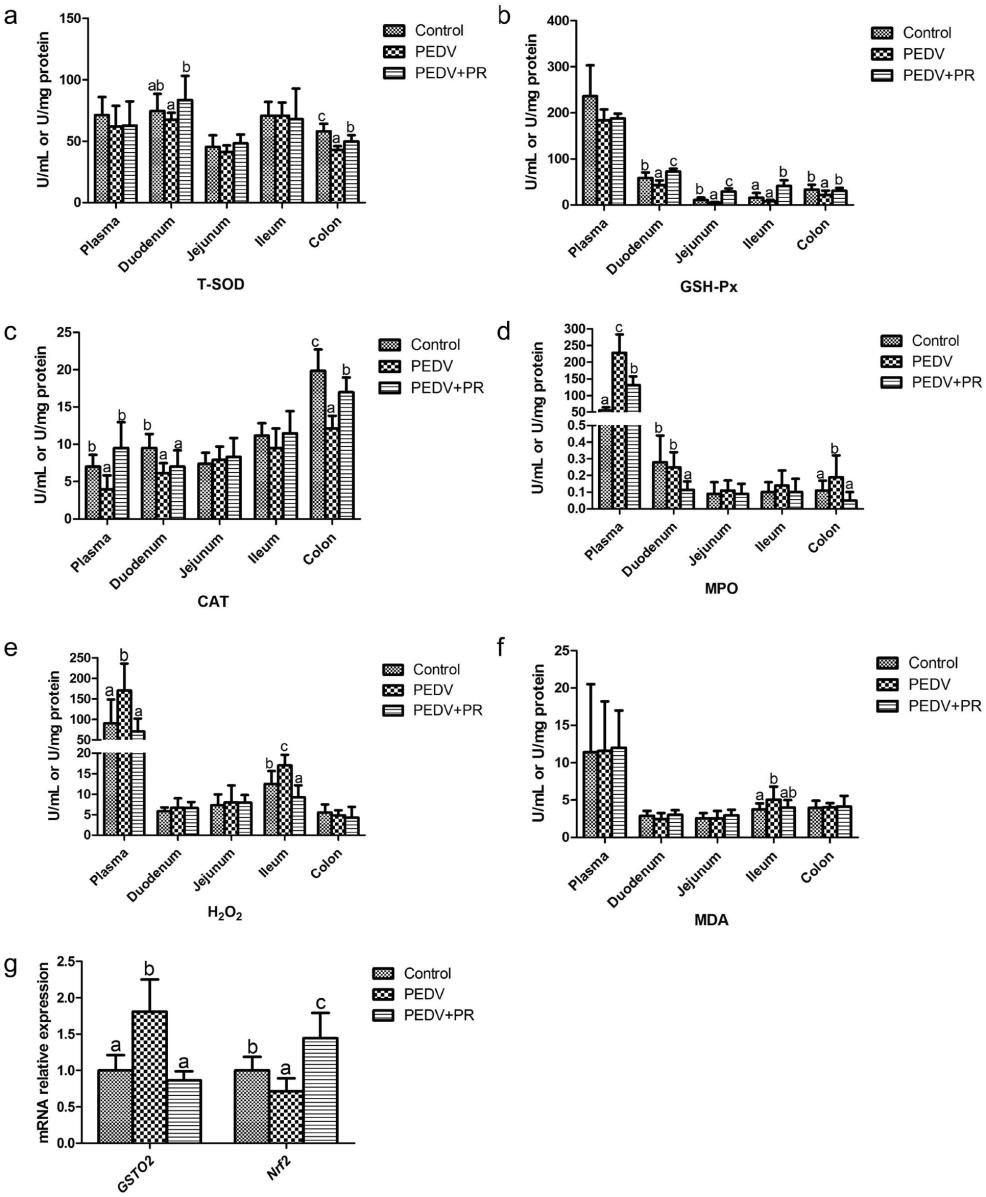
Effects of puerarin (PR) administration on the redox status of piglets after PEDV infection. (**a**) T-SOD, total superoxide dismutase, (**b**) GSH-PX, glutathione peroxidase, (**c**) CAT catalase, (**d**) MPO myeloperoxidase, (**e**) H_2_O_2_, hydrogen peroxide, (**f**) MDA malondialdehyde, (**g**) *GSTO2*, Glutathione S-transferase omega 2; *Nrf2*, Nuclear factor erythroid 2-related factor 2 *.* Values are mean and SD, *n* = 8. ^a, b, c^, Values within a column not sharing a common superscript letter indicate significant difference at *P* < 0.05.

Data on activities of T-SOD, GSH-PX, CAT and MPO, as well as the concentrations of MDA and H_2_O_2_ are summarized in Fig. 3. Compared with the control, PEDV infection reduced the activities of CAT in the plasma, duodenum and colon, T-SOD in the colon, and GSH-Px in the duodenum, jejunum and colon, but increased the activities of MPO in the plasma and colon, and the concentrations of MDA in the ileum and of H_2_O_2_ in the plasma and ileum (*P* < 0.05). However, compared with the PEDV group, the PEDV + PR group had higher activities of T-SOD in the duodenum and colon, GSH-Px in the duodenum, jejunum, ileum and colon, and CAT in the plasma and colon, but lower levels of MPO in the plasma, duodenum and colon, as well as lower levels of H_2_O_2_ in the plasma and ileum (*P* < 0.05). Additionally, as shown in Fig. 3, compared with the control, PEDV infection increased the mRNA levels of *GSTO2*, but reduced the mRNA levels of nuclear factor carotenoid 2 related factor 2 (*Nrf2*). In contrast, PR administration decreased the mRNA levels of *GSTO2*, but increased the mRNA levels of *Nrf2.*

### Concentration of cytokines in plasma

**Figure 4:**
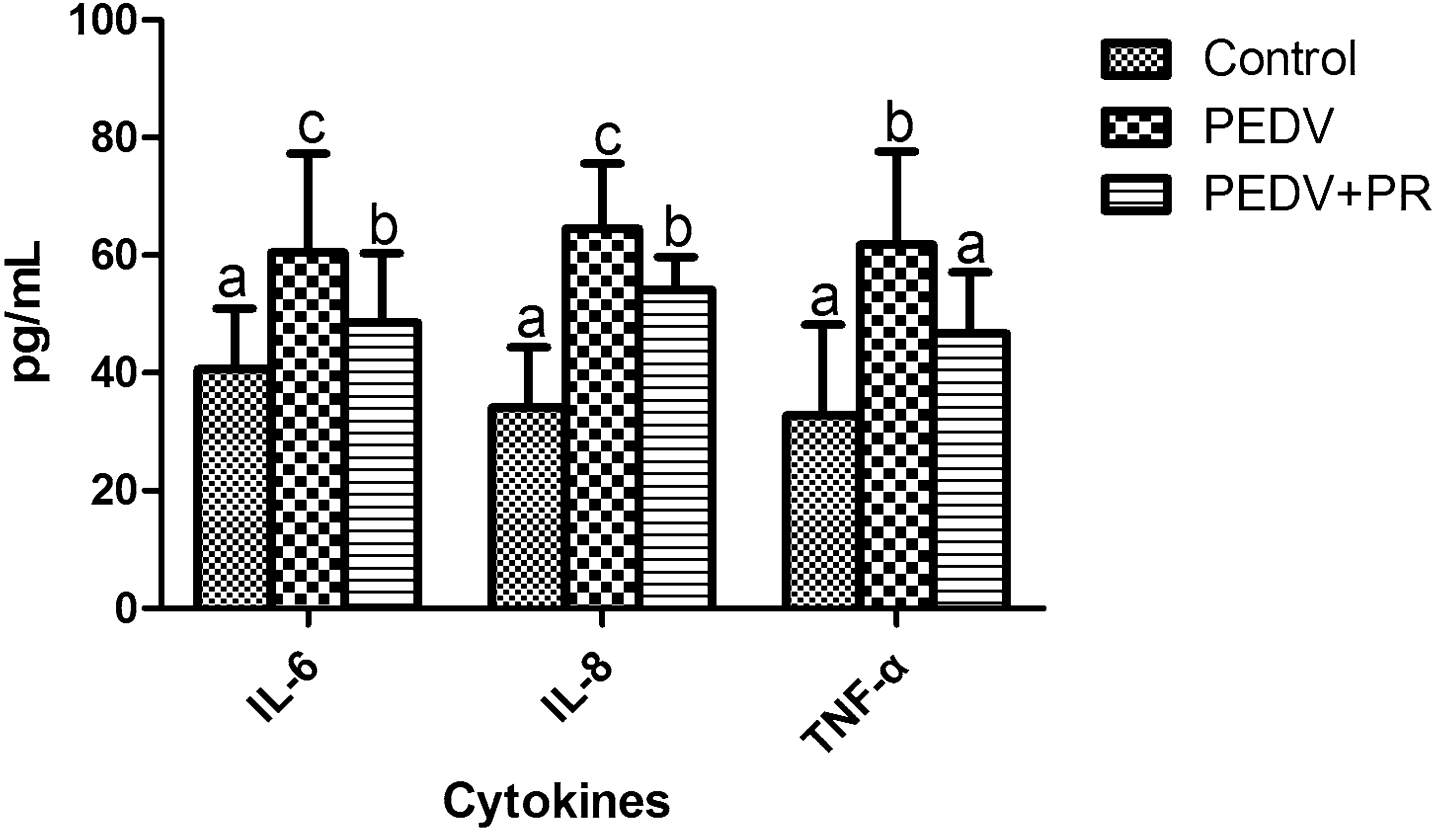
Effects of puerarin (PR) administration on cytokines in the plasma of piglets after PEDV infection. Values are mean and SD, *n* = 8. ^a, b, c^, Values within a column not sharing a common superscript letter indicate significant difference at *P* < 0.05.

PEDV infection increased the concentrations of IL-6, IL-8, and TNF-α in plasma, as compared with the control (*P* < 0.05). However, PR intervention reduced the concentrations of IL-6, IL-8, and TNF-α in the plasma of the PEDV-infected piglets (*P* < 0.05). The results are shown in Fig. 4.

### HSP70 and villin protein abundances

**Figure 5:**
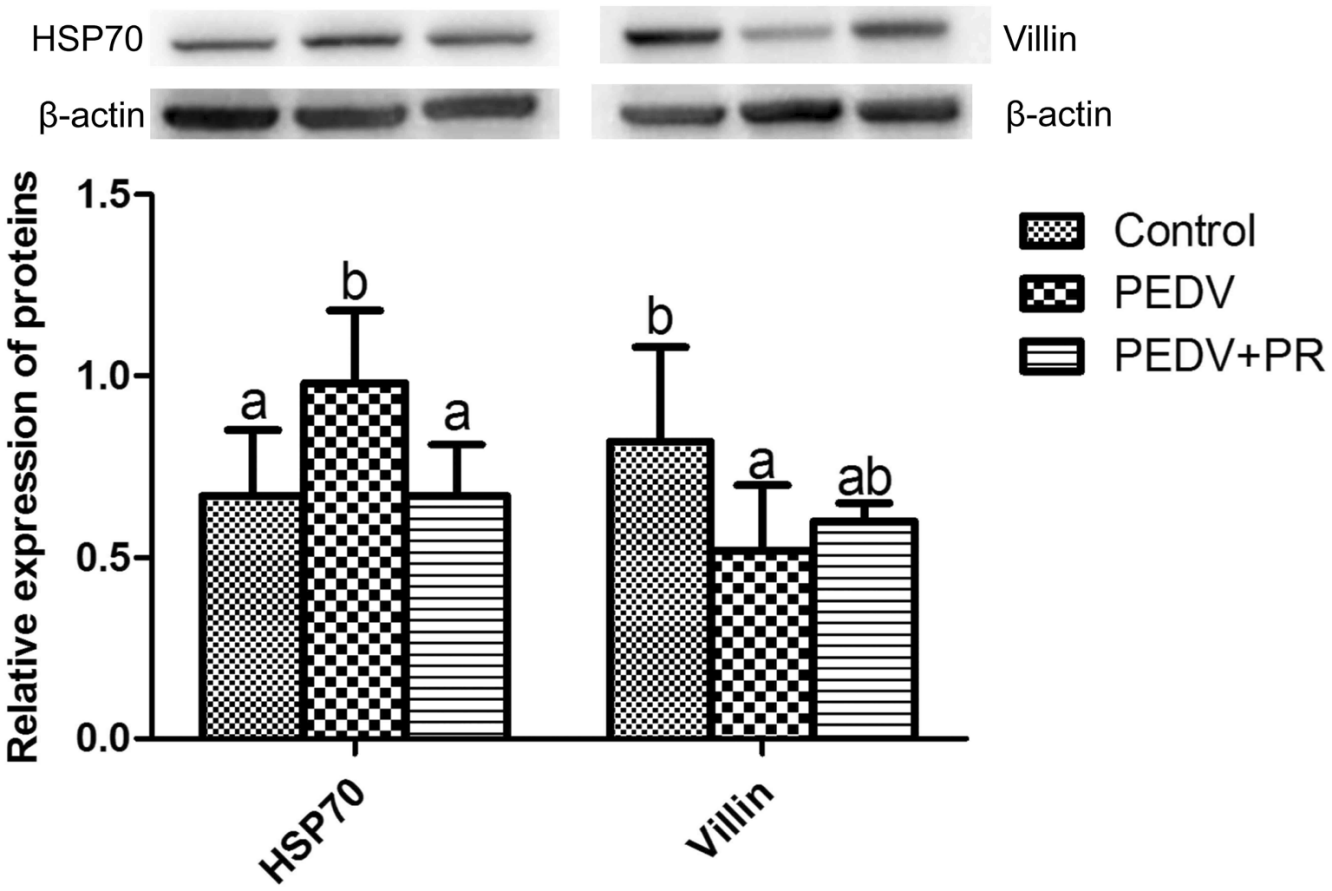
Effects of puerarin (PR) administration on relative expression of HSP70 and Villin in ileum tissue of piglets after PEDV infection. Values are mean and SD, *n* = 8. ^a, b, c^, Values within a column not sharing a common superscript letter indicate significant difference at *P* < 0.05.

PEDV infection significantly increased the abundance of the HSP70 protein, but decreased the abundance of the villin protein, compared to the control (*P* < 0.05). However, PR administration decreased the abundance of the HSP70 protein (*P* < 0.05) in comparison with the PEDV group (Fig. 5).

### Abundance of selected intestinal bacteria

**Figure 6:**
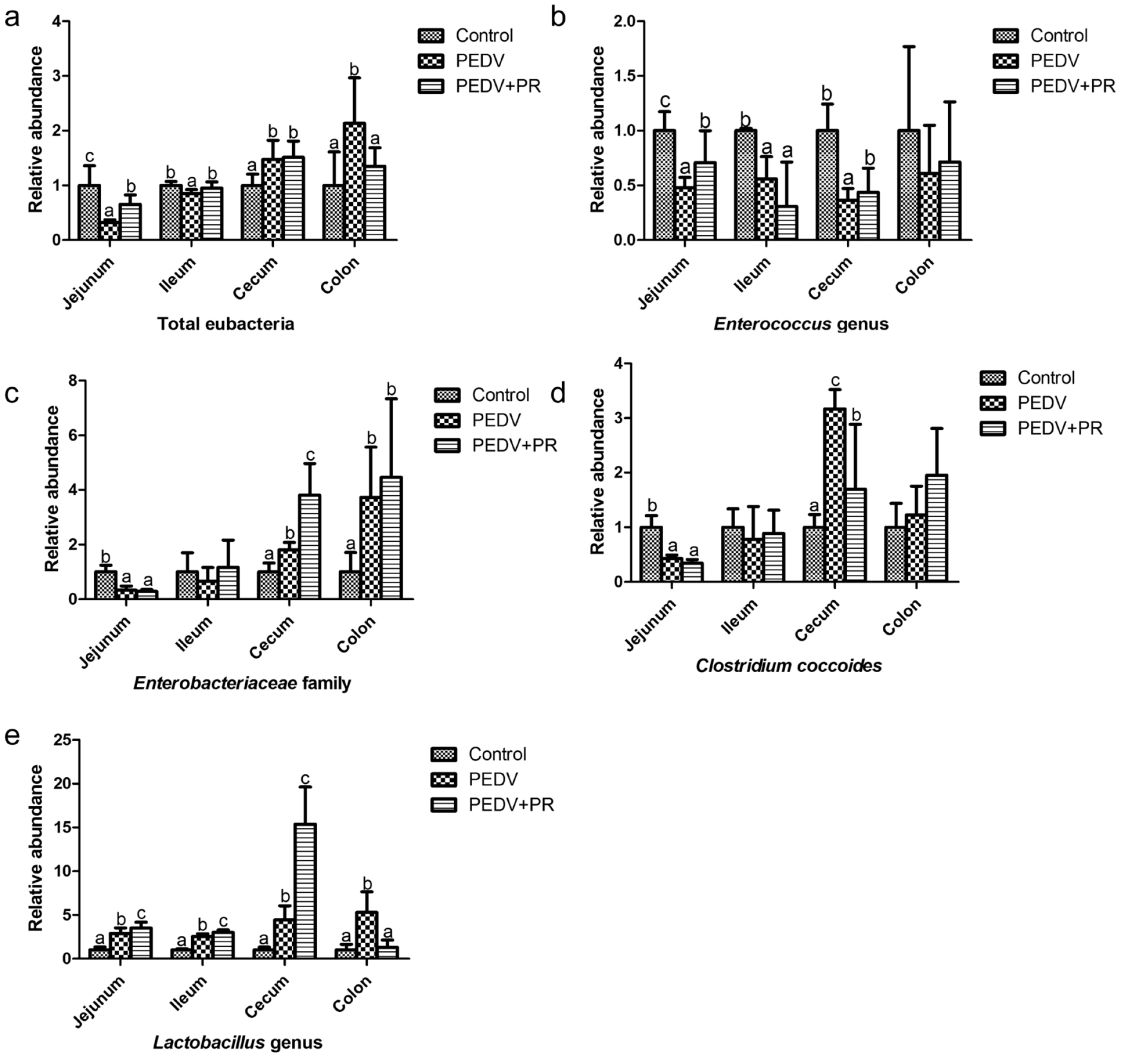
Effects of puerarin (PR) administration on selected bacteria of piglets after PEDV infection, (**a**) Total eubacteria (16S rRNA)*,* (**b**) *Enterococcus* genus, (**c**) *Enterobacteriaceae* family, (**d**) *Clostridium coccoides*, (**e**) *Lactobacillus* genus Values are mean and SD, *n* = 8. ^a, b, c^, Values within a column not sharing a common superscript letter indicate significant difference at *P* < 0.05.

Compared with the control, PEDV infection reduced the number of *Enterococcus* genus*,* but increased the number of *Lactobacillus* genus in the jejunum, ileum, caecum and colon*.* Moreover, PEDV infection reduced the abundances of total eubacteria in the jejunum and ileum, *Clostridium coccoides* and *Enterobacteriaceae* family in the jejunum, while increasing the abundances of total bacteria in the caecum and colon, *Clostridium coccoides* in caecum and *Enterobacteriaceae* family in the caecum and colon (*P* < 0.05). Compared with the PEDV group, PR administration increased the abundances of *Enterococcus* genus in the jejunum and caecum, *Lactobacillus* genus in the jejunum, ileum and caecum, total bacteria in the jejunum and ileum*, Enterobacteriaceae* family in the caecum, but reduced the abundance of *Lactobacillus* genus and total eubacteria in the colon, and *Clostridium coccoides* in the caecum (*P* < 0.05) (Fig. 6).

## Discussion

PEDV infection is a significant challenge to swine production worldwide^9–11^. Its clinical symptoms include diarrhea and vomiting, as well as reductions in body weight. Results of our study indicated that PR intervention reduced morbidity in infected piglets, as compared with the PEDV group without PR administration. As an antioxidant phytochemical, PR could be used as herbal medicine to inhibit intestinal inflammation and improve intestinal function. This can be translated into sustaining global pig production.

The immune system status of animals could be evaluated by the numbers of white blood cells, including neutrophils, lymphocytes, and monocytes^19,20^. These cells play a key role in the process of inflammation and tissue damage. Recruitment or extravasation of leukocytes is their migration toward an area of inflammation, injury, or infection^21^. There are pieces of evidence that blood lymphocytes are significantly elevated after PEDV infection or vaccination^22,23^. Infiltration of lymphocytes, eosinophils, neutrophils and other mononuclear cells were found in the lamina propria of the small intestine after PEDV infection^24^. In good agreement with these studies, our results demonstrated that the numbers of blood WBC, NEU and LYM significantly increased in piglets after PEDV infection. Monocytes are a subset of circulating white blood cells that can further differentiate into tissue macrophages and dendritic cells (DCs)^25^. Recruitment of monocytes is essential for mediating the host antimicrobial defense and is also implicated in many inflammatory diseases^26^. PEDV infection of neonatal piglets triggered a strong and rapid induction in type I interferon^27^. DC is known to secret cytokines such as type I interferon, IL-12, IL-10, and chemokine to regulate the subsequent immune responses^28^. In the present study, the decrease of blood monocytes in piglets after acute PEDV infection may be that monocytes are rapidly recruited to the intestinal injury site and differentiated into dendritic cells to participate in the immune response. More studies are needed to test this hypothesis. One study has shown the upregulation of genes for IL-1β, IL-6, IL-8, and TNF-α in IPEC-J2 cells infected with PEDV^29^. Consistently, we previously reported that the mRNA levels of related inflammatory factors in Vero cells and the levels of inflammatory factors in the ileal tissue are elevated in PEDV-challenged piglets^8^. However, blood circulation is vital in the development of systemic inflammation. In the current study, the concentrations of pro-inflammatory cytokines, such as IL-6, IL-8, and TNF-α, were increased in the plasma of PEDV-infected pigs. Taken it together, these findings indicated that PEDV infection induced systemic inflammation in piglets. Notably, we found that PR administration significantly decreased the numbers of immune cells and the concentrations of blood pro-inflammatory cytokines in PEDV-infected piglets, thereby potentially mitigating inflammation and tissue injury in the animals. Our results are consistent with those that PR exerted protective effects against neutrophil infiltration in rats^30^, decreased neutrophil adhesion in cultured endothelial cells^31^, and prevented LPS-induced acute injury in mice^32^ and rats^33^. Therefore, PR may attenuate systemic inflammation by suppressing the production of pro-inflammatory mediators.

Another indicator of tissue damage is the levels of enzymes in plasma. For example, abnormal hepatic biochemical parameters in patients with inflammatory bowel disease (IBD) are associated with increased activities of AST, ALT and ALP in plasma^34^. In the present study, the decreases in plasma AST and ALP levels after PR intervention provided further evidence for the anti-inflammatory effect of PR. Additionally, AST, ALT, AST/ALT ratio, and ALP in plasma are good indicators of liver function^35–37^. Furthermore, one study found that PEDV-induced up-regulated proteins were involved in 22 diseases and disorders, such as hepatic diseases, gastrointestinal diseases, and inflammatory responses^9^. It appears that PR could attenuate the PEDV-induced liver dysfunction because PR administration attenuated the rise in plasma AST, AST/ALT ratio and ALP in PEDV-infected piglets. The underlying mechanisms are largely unknown and warrant to be elucidated. PEDV damage the pig intestinal epithelium, leading to reduced integrity and function of the mucosal barrier^38^. Intestinal mucosal barrier function can be commonly assessed by several indicators, including D-xylose concentration and DAO activity in plasma^39,40^. DAO is a highly active intracellular enzyme produced by the intestinal epithelial cells and only presents in the intestinal mucosa and ciliated cells^41^. In the case of malabsorption, the entry of D-xylose from the intestinal cavity to the portal vein is destructed, thereby reducing the concentration of D-xylose in the blood. Therefore, plasma D-xylose level and DAO activity can be used as indicators of intestinal absorption function and integrity^42^. Additionally, among the FABP family proteins, I-FABP is particularly abundant in epithelial cells of the small intestinal mucosa and it is rapidly released into the circulation when the small intestinal mucosa is impaired. Therefore, I-FABP could be used as a tissue-specific injury marker^43,44^. In our previous study, histologic lesions were present in the jejunum and ileum of PEDV-infected piglets, including severe villous atrophy^8^. In the present study, PEDV decreased plasma D-xylose and I-FABP concentrations, while increasing DAO activity in the plasma, indicating that PEDV induced intestinal dysfunction. Importantly, PR administration enhanced intestinal function, as indicated by an increase in the plasma concentrations of D-xylose and I-FABP and a decrease in plasma DAO activity. Previous studies also reported that PR could improve intestinal mucosal barrier function in mice^45,46^. Furthermore, we found that PR administration attenuated the PEDV-induced decrease in the expression of villin, a marker of villus cell differentiation in ileal mucosae^47,48^. Based on molecular level, detection indicators, the current results further support our previous conclusion. Taken together, these data indicated that PR could alleviate intestinal injury and improve intestinal function in PEDV-infected piglets.

Hydrogen peroxide (H_2_O_2_) and malondialdehyde (MDA) levels in the intestinal mucosa and plasma are important indicators of oxidative stress^49^. Neutrophil myeloperoxidase (MPO) is a rich granule enzyme that catalyzes the production of potent ROS, the latter serves as a biomarker for oxidative damage and is raised in patients with inflammatory bowel disease (IBD)^50^. Glutathione S-transferase is also an important biomarker for inflammation and oxidative stress^51^. GSTO2 plays a protective role in counteracting oxidative stress^52^. In the present study, the levels of MDA, H_2_O_2,_ MPO and GSTO2 were elevated but CAT, T-SOD and GSH-Px activities in the plasma and intestine were decreased in PEDV-infected pigs. These results were consistent with our previous report that supplementation with N-acetylcysteine (an antioxidant) alleviated intestinal injury in piglets infected by PEDV^53^. In addition, the expression of HSP70, one of the most important cellular defense mechanisms, is induced under stressful conditions such as infection^54^. Interestingly, HSP70, GSH-Px and total antioxidant capacity are significantly up-regulated in pigs and rats after PEDV infection^55–57^. A study found that Nrf2 may increase the expression of antioxidant related genes by acting on the antioxidant response element (ARE) in the gene promoter, therefore control abnormal oxidative stress^58^**.** In the present study, PR increased the mRNA levels of *Nrf2* under PEDV infection. The result was similar to previous studies demonstrating that Nrf2 is a key regulator on puerarin preventing oxidative stress damage in rodents^59–61^. Collectively, our results indicated that PR could effectively improve the redox status and alleviate oxidative damage in piglets infected with PEDV.

The intestinal microbiota is important for gastrointestinal function and health. Dynamic changes in the gut microbiota of piglets may contribute to decreases in morbidity and mortality in the older PEDV-infected piglets^62^. In the current study, although *Enterobacteriaceae* family and *Clostridium coccoides* were reduced in the jejunum, they were increased in the caecum and colon after PEDV infection. *Enterobacteriaceae* family is commonly present in the gastrointestinal tract and represents a group of potentially pathogenic microflora^63^. One study observed a statistically significant predominance of *Clostridium_Sensu_Stricto*_1 in 2-week-old piglets after PEDV infection^64^. *Enterococcus* genus, which has been studied as a probiotic^65^, was reduced in the jejunum, ileum and cecum after PEDV infection. *Lactobacillus* genus is commonly investigated as a probiotic agent^66^. *L. johnsonii*, one of the *lactobacilli*, has been shown to enhance the resistance of cells to *E. coli* challenge and can be used as a probiotic to handle the problem of piglet diarrhea^67,68^. However, another study found *Lactobacillus* was elevated in children diagnosed with irritable bowel syndrome^69^. It is still unclear whether the increase of *Lactobacillus* in the intestine could protect animals against PEDV. Another study found that total bacteria in the small intestine was reduced after transmissible gastroenteritis virus (TGEV) infection in swine^70^, which was consistent with our results. However, the increase of total eubacteria in the large intestine may be attributed to the increase of *Lactobacillus* genus*, Enterobacteriaceae* family*,* and *Clostridium coccoides.* Consistent with this notion, PR can be used as a preventive treatment for *Clostridium difficile-*associated diarrhea in a mouse model^71^. A lower abundance of *Clostridium coccoides* was detected in the caecum, while a higher abundance of the *Enterococcus* genus in the jejunum and caecum after PR intervention. Intriguingly, there is a significant increase in the number of *Lactobacillus* genus in the PEDV + PR group as compared with the PEDV group and, even to a greater extent with the control group. Collectively, PR intervention could maintain the balance of intestinal microflora and increase the number of beneficial bacteria. Considering the reduced morbidity, the improvements of intestinal mucosal barrier function indicators and anti-oxidative function, we believe that the changes in the abundance of selected microorganisms in the gut in response to PR intervention are beneficial for intestinal health in PEDV-infected piglets. Because the digestion, absorption and metabolism of nutrients are crucial for animal growth and survival^72^, regulating the intestinal bacteria may be an alternative measure to prevent or treat PED. Further researches are necessary to identify specific changes in the intestinal flora at the species level, especially the *Lactobacillus* genus. This is the first time to report the effect of PR on anti-oxidation and some specific bacteria in the intestine of PEDV-infected piglets.

## Conclusion

Oral administration with PR reduced morbidity in piglets infected with PEDV. The beneficial effects of PR on intestinal function were associated with the following: (1) enhanced anti-inflammatory functions (indicated by the decreases in AST, ALP, immune cell numbers and cytokines levels); (2) improved anti-oxidative capacity (indicated by improving redox status and attenuating oxidative damage); (3) possibly enhanced intestinal mucosal barrier; and (4) increased the abundance of intestinal beneficial bacteria. These results provide important insights into the development of effective prevention against PEDV infection and other enteric diseases.

## Materials and methods

### PEDV and puerarin

PEDV (Yunnan province strain, GenBank accession No. KT021228) was kindly provided by State Key Laboratory of Agricultural Microbiology, College of Veterinary Medicine, Huazhong Agricultural University (Wuhan, China). PR (purity ≥ 98%) was purchased from Macklin (Macklin Inc., Shanghai, China).

### Animals and treatments

Twenty-four crossbred (Duroc × Landrace × Large White) 7-day-old healthy piglets, half male and half female with similar body weight (3.17 ± 0.51 kg), were randomly divided into one of three groups (Control, PEDV, and PEDV + PR) with 8 replicates per group. All pigs were purchased from a PEDV-free farm. These piglets were not vaccinated with a PEDV vaccine. The experiment lasted for 12 days. During day 5 to day 9 of the trial, piglets in the PEDV + PR group were orally administered with PR at the dosage of 0.5 mg/kg body weight (dissolved in a liquid milk replacer), whereas the other two groups were given the same volume of liquid milk replacer. The dose of PR was chosen according to our preliminary study that PR (0.5 mg/kg BW) could exert antiviral and anti-inflammatory effects in piglets infected with PEDV^8^. On day 9 of the trial, piglets in the PEDV and PEDV + PR groups were orally inoculated with 3.3 mL of PEDV solution at 10^4.5^ TCID_50_ (50% tissue culture infectious dose) per pig, while those in the control group were orally inoculated with an equal volume of sterile saline. D-xylose (0.1 g / kg body weight) was orally administered to all pigs on day 12 of the trial. One hour later, blood samples were obtained from the jugular vein, and thereafter all piglets were weighed and sacrificed by injection of sodium pentobarbital (50 mg/kg Body weight) to collect intestinal tissues and chyme as described previously^53,73^. Briefly, the intestine was dissected free of the mesentery tissue, and segments were obtained from the distal duodenum, mid-jejunum, mid-ileum, mid-caecum, and mid-colon, respectively. Intestinal contents were collected carefully and then intestine was flushed with ice-cold PBS. The mucosa was collected through scraping using a sterile glass microscope slide. All the sampling procedures were conducted at a chilled glass plate. All samples were stored at -80 ℃ until further analysis. The experimental basic feed (liquid milk substitute) formulated to meet all the nutrients required by suckling pigs was purchased from Wuhan Anyou Feed Co., Ltd. (Wuhan, China). Piglets were housed in clean pens with the strict prevention of cross-infection. The control group was quarantined from the infection groups and in different rooms. Piglets were observed daily and weighed to analyze the health status (body weight, morbidity). For the assessment of morbidity, clinical signs were recorded in all piglets, including diarrhea (defined as any loose or soft stool observed during defecation or seen on the piglets), dehydration (defined as sunken eyes and the appearance of wrinkled skin), and vomiting^74^. The specific evaluator was blind to the experimental design and objectively registered the signs. The animal use protocol for the present study was approved by the Animal Care and Use Committee of Wuhan Polytechnic University (Index number: 011043145–029-2013–000,009).

### Plasma biochemical parameters and blood cell counts

The method was presented in the previous study^53^. In short, one hour after oral administration of D-xylose on day 12 of the experiment, blood samples were collected from the anterior vena cava into heparinized vacuum tubes (Becton–Dickinson Vacutainer System, Franklin Lake, NJ, USA) and then centrifuged at 3000 rpm for 10 min at 4 ℃ to obtain plasma. Plasma biochemical parameters were measured according to the manufacturer’s instructions by a Hitachi 7060 Automatic Biochemical Analyzer (Hitachi, Japan)^75^. Blood cell counts were performed on the Siemens ADVIA 2120i Hematology Analyzer (Siemens Healthcare Diagnostics, Deerfield, Illinois, USA).

### Determination of D-xylose and diamine oxidase (DAO) in plasma

The kit for detecting D-xylose in plasma was purchased from Nanjing Jiancheng Bioengineering Institute (Nanjing, China), and operated following the procedure described previously^76^. 50 μL of the plasma was added to 5 mL of the phloroglucinol color reagent solution (Sigma Chemical Inc., St. Louis, MO, USA), then heated at 100 ℃ for 4 min in a water bath. After the samples were cooled to room temperature, the absorbance of the mixed solution at 554 nm was measured using a SpectraMax i3x Multi-Mode Detection Platform (Molecular Devices, LLC, Sunnyvale, CA, USA). The standard solution of 0 mmol/L D-xylose was considered as the blank. In addition, DAO activities in plasma were determined by using spectrophotometry according to the manufacturer’s instructions^77^. The assay kit was purchased from Nanjing Jiancheng Bioengineering Institute (Nanjing, China).

### Activities of anti-oxidant enzymes and levels of oxidation-relevant products in plasma and intestinal mucosae

The activities of catalase (CAT), myeloperoxidase (MPO), total superoxide dismutase (T-SOD) and glutathione peroxidase (GSH-Px) and the concentrations of hydrogen peroxide (H_2_O_2_) and malondialdehyde (MDA) were determined by using commercially available kits (Nanjing Jiancheng Bioengineering Institute, Nanjing, China) according to the manufacturer’s protocols. Assays were performed in triplicate.

### Intestinal fatty acid-binding protein (I-FABP) and cytokine determination by enzyme-linked immunosorbent assay (ELISA)

Concentrations of I-FABP and cytokines (IL-6, IL-8 and TNF-α) in the plasma were measured by using commercial ELISA kit (R&D Systems, CA, USA), which were performed according to the manufacturer’s instructions.

### Western blot analysis

Western blot was performed according to the previous method^78^. In brief, proteins were extracted from intestinal mucosae and the concentrations were determined by using the bicinchoninic acid assay (Thermo Scientific, USA). Equivalent quantities of proteins from the independent biological replicates were denatured in 5 × sample loading buffer by heating at 100 ℃ for 5 min and separated by 10% SDS-PAGE. Separated proteins were then electrophoretically transferred onto polyvinylidene difluoride (PVDF) membrane and blocked with 5% w/v skim milk in Tris-buffered saline containing Tween 20 (TBST) for 1.5 h at room temperature. Membranes were incubated with primary antibodies at 4 ℃ overnight: heat shock protein 70 (HSP70, Enzo, 1: 1000), villin (Santa, 1: 1000). After washed three times with TBST, membranes were incubated with the anti-rabbit (mouse) immunoglobulin G horseradish peroxidase conjugated secondary antibody (Beijing ZhongShan Golden Bridge Biological Technology Co. Ltd, Beijing, China; 1:5000 dilution). After being washed with TBST, blots were detected by enhanced chemiluminescence Western blotting kit (ECL-plus, Amersham Biosciences, Sweden). β-actin (Invitrogen, 1:4000) was determined as an internal reference.

### Quantitative RT-PCR (qRT-PCR) and droplet digital PCR (dd PCR)

The total RNA in ileum tissue was extracted by TRIzol reagent (Takara, Dalian, China) to ensure the purity (a 28 S/18 S rRNA ratio of > 1.8 and an OD_260_/OD_280_ ratio of approximately 2.0). The cDNA was synthesized by RT-PCR using the PrimeScript RT reagent Kit with gDNA Eraser (Takara, Dalian, China) according to the manufacturer’s instructions. The qPCR was carried out by using the SYBR Premix Ex Taq (Takara, Dalian, China) on an Applied Biosystems 7500 Fast Real-Time PCR System (Foster City, CA). The relative expression level of each gene was calculated with the 2^−ΔΔCT^ method^79^. Ribosomal protein L4 (*RPL4*) was used as the reference gene in the ileum.

Genomic DNA was extracted from chyme by using QIAamp Fast DNA Stool Mini Kits (Qiagen, Hilden, Germany) according to the manufacturer’s instructions and the concentration was measured using a NanoDrop 2000 spectrophotometer (Thermo Scientific, USA). The ddPCR was conducted in a QX200 Droplet Digital PCR system (Bio-Rad) as described previously^80^. In short, the final volume of each assay mixture was 20 μL, which contained 10 μL of 2x ddPCR supermix,100 nM primers and 4 μL of extracted DNA. Then the QX200 Droplet Generator (Bio-Rad) was used to generate the droplets according to the manufacturer’s instructions. PCR amplification was performed using the following conditions: 1 cycle of 95 ˚C for 10 min, 40 cycles of 94 ˚C for 30 s, 55 ˚C for 1 min, and 98 ˚C for 10 min. The droplets were quantified by QX200 Droplet Reader (Bio-Rad). Since no standard curve was required for the ddPCR^81^, therefore, the fluorescent signal events above the threshold line were analyzed by QuantaSoft Software (Bio-Rad). According to preliminary experiments, the threshold of 10,000 was selected to separate positive and negative droplets. The universal primers were used to analyze total eubacteria. Specific 16S rRNA genes were targeted for these selected bacteria (*Enterococcus* genus*, Enterobacteriaceae* family*, Clostridium coccoides* and *Lactobacillus* genus). The relative abundance levels of genes in the treatment groups were normalized to the control group to determine the differences among the groups, as we described previously^73^. Primers used in the present study are listed in Table 2.

**Table 2:** The sequences of primers used in the present study.

Gene name	Sequence	References
RPL4	F:5′-GGAAACCGTCGCGAGA-3'	^8^
	R:5′-GCCCCAGAGACAGTT-3'	
GSTO2	F:5′-GCCTTGAGATGTGGGAGAGAA-3'	^82^
	R:5′-AAGATGGTGTTCTGATAGCCAAGA-3'	
Nrf2	F:5′-ATCACCTCTTCTGCACCGAA-3'	Present study
	R:5′-GCTTTCTCCCGCTCTTTCTG-3'	
*Enterobacteriaceae* family	F:5′-CATTGACGTTACCCGCAGAAGAAGC-3'	^73^
	R:5′-CTCTACGAGACTCAAGCTTGC-3	
*Enterococcus* genus	F:5′- CCCTTATTGTTAGTTGCCATCATT-3'	^73^
	R:5′-ACTCGTTGTACTTCCCATTGT-3'	
*Clostridium coccoides*	F:5′-AATGACGGTACCTGACTAA-3'	^73^
	R:5′- CTTTGAGTTTCATTCTTGCGAA-3'	
*Lactobacillus* genus	F:5′-AGCAGTAGGGAATCTTCCA-3'	^73^
	R:5′-CACCGCTACACATGGAG-3'	
Total eubacteria (16S rRNA)	F:5′-CGGTCCAGACTCCTACGGG-3'	^73^
	R:5′-TTACCGCGGCTGCTGGCAC-3'	

RPL4 ribosomal protein L4.

GSTO2 glutathione S-transferase omega 2.

Nrf2 nuclear factor erythroid 2-related factor 2.

### Statistical analysis

Data were reported as means with SD and were analyzed by one-way ANOVA in the SPSS 17.0 statistical software (SPSS Inc. Chicago, USA). The data of the morbidity was analyzed by χ2 analysis and expressed as a percentage. Following the 2^−ΔΔCt^ method, the mean value of the ileal gene expression of piglets in the control group was set to 1.00. The mean value of the relative abundance of bacteria in the control group was also set to 1.00. Multiple comparisons of means by the Duncan test was performed when the difference is significant. P-values ≤ 0.05 were taken to indicate statistical significance.

### Ethics statement

All animal works were conducted according to the guidelines for the care and use of experimental animals approved by the Animal Care and Use Committee of Wuhan Polytechnic University (Index No.: 011043145-029-2013-000009) and the ARRIVE guidelines.

## Supplementary Information

Supplementary Information

## Data Availability

We declare that we support data availability, which allows unlimited access to our published materials, data and associated protocols promptly available to readers.

## Abbreviations

CAT: Catalase
DAO: Diamine oxidase
GSH-Px: Glutathione peroxidase
H_2_O_2_: Hydrogen peroxide
I-FABP: Intestinal fatty acid-binding protein
IL-6: Interleukin-6
IL-8: Interleukin-8
MDA: Malondialdehyde
MPO: Myeloperoxidase
PEDV: Porcine epidemic diarrhea virus
SD: Standard deviation
TCID_50_: 50% Tissue culture infectious dose
TNF-α: Tumor necrosis factor-α
T-SOD: Total superoxide dismutase
