# Supplementary material for: Puerarin enhances intestinal function in piglets infected with porcine epidemic diarrhea virus
Source: Sci Rep. 2021 Mar 22;11:6552. doi: 10.1038/s41598-021-85880-5 (PMC7985190; doi:10.1038/s41598-021-85880-5)

Puerarin enhances intestinal function in piglets infected with porcine epidemic diarrhea virus

Mengjun Wu[1,3†], Dan Yi[1†], Qian Zhang[1†], Tao Wu[1], Kui Yu[1], Meng Peng[1], Lei Wang[1], Di Zhao[1], Yongqing Hou[1*], and Guoyao Wu[2]

[1]Hubei Key Laboratory of Animal Nutrition and Feed Science, Wuhan Polytechnic University, Wuhan, China 430023
[2]Department of Animal Science, Texas A&M University, College Station, Texas, USA 77843
[3]Department of Agricultural, Environmental and Food Sciences, University of Molise, Campobasso, Italy 86100

* Correspondence:
Yongqing Hou, Ph. D
Hubei Key Laboratory of Animal Nutrition and Feed Science,
Wuhan Polytechnic University, Wuhan 430023, China
E-mail address: houyq@aliyun.com

† These authors contributed equally to this work and should be considered co-first authors

Supplementary Material 1-original images of the western blot

Figure 5

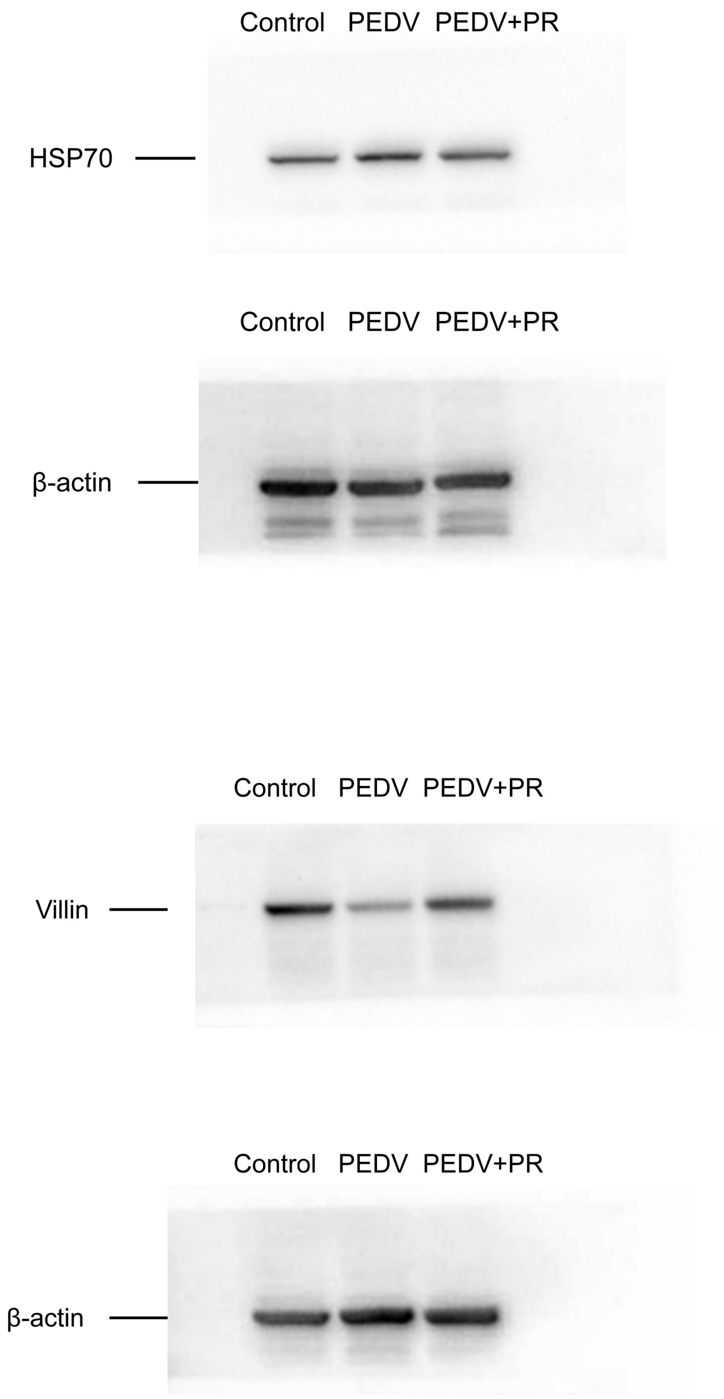

Supplement: Supplementary file 1 — Supplementary Information [file 41598_2021_85880_MOESM1_ESM.pdf]
